# Supplementary material for: An Integrated Approach to Assess the Environmental Impacts of Large-Scale Gold Mining: The Nzema-Gold Mines in the Ellembelle District of Ghana as a Case Study
Source: Int J Environ Res Public Health. 2021 Jul 1;18(13):7044. doi: 10.3390/ijerph18137044 (PMC8295839; doi:10.3390/ijerph18137044)
Supplement: Supplementary file 1 [file ijerph-18-07044-s001.zip › ijerph-1246742-supplementary.pdf]

<u>Questionnaire to EPA and District Environmental Officer</u>

TOPIC: Assessing the Environmental Impacts of Large-Scale Gold Mines, the Case of Nzema Gold Mines in the Ellembelle District of Ghana.

Note: ***This questionnaire is meant for academic and publications purposes with anonymity seriously observed.***

Questionnaire to help determine the Impacts of the activities of **LARGE SCALE** Gold mines on the environment of hosting communities (Nkroful, Anwia, T. Bokazo and Salman) and the District in general. The questionnaire is based on the following key elements of the environment: Water, Climate, Vegetation/Biodiversity, Air, Noise, and Soil/Land.

Kindly tick in the boxes and fills the spaces provided.

Age ………………   Gender ……………………..

Kindly tick in the boxes and fills the spaces provided.

1. How many large-scale mining companies operate the Nzema gold mines? ……………… and their name(s)………
2. What are the major water bodies (rivers and streams) around mining sites?
   ……………………………………………………………., ……………………………………………………………….
   ……………………………………………………………., ……………………………………………………………….
   ……………………………………………………………., ………………………………………………………………
   ……………………………………………………………., ………………………………………………………………..
3. Which of these water bodies is/are affected by mining activities?
   …….
4. How are these water bodies affected by mining activities?
   …….
   …….
   …….
   …….

5. Does mining company(s) directly pump water from any of the available rivers for it operations? **Yes[ ]** **No[ ]**

   If yes, which river(s)……………………………………………………………………………………………………

6. Has there been any tailings dam (if any) leakage or seepage? **Yes[ ]** **No[ ]**
7. Has mining operations affected the climatic conditions of the District or host communities? **Yes[ ]** **No[ ]**
8. Does mining operations affect the rainfall pattern of the District? **Yes[ ]** **No[ ]**
9. If yes**,** how is the rainfall pattern affected? ***(Please tick more than one if appropriate).***
   i) Increased in annual rainfall **[ ]**
   ii) Decreased in annual rainfall **[ ]**
   iii) Constant changes in rainfall seasons **[ ]**
10. Does mining operations affect the temperature of the host communities? **Yes[ ]** **No[ ]**
11. If yes, how is temperature affected? ***(Please tick more than one if appropriate).***
    i) Increasing/ high temperature over the year (annual) **[ ]**
    ii) Decreasing/ low annual temperature **[ ]**
    iii) Constant variations in annual temperature **[ ]**
12. Has the mining activities or operations caused loss of vegetation cover? **Yes[ ]** **No[ ]**
13. What are the noticed negative effects of vegetation loss on lands and environment of host communities?

    ……

    ……

    ……

    ……

14. Are there abandoned mining sites in the district? **Yes[ ]** **No[ ]**
15. If yes, have there been afforestation or reclamation programs in abandoned mining sites? **Yes[ ]** **No[ ]**
16. Has the emergence of the mines caused changes in land-use patterns in hosting communities? **Yes[ ]** **No[ ]**
17. Does mining activities have effects on soil quality? **Yes[ ]** **No[ ]**

18. If yes, how is the soil quality being affected by mining activities?

………

………

………

………

19. What is the PH level of soil in the hosting communities?

Nkroful………………, Anwia………………, T.Bokazo……………………, Salman……………………

20. Has there changes in PH levels of soil as a result of the mining operations? **Yes[ ]** **No[ ]**

21. How does mining companies manage their liquid waste?

    i. Discharge to water bodies **[ ]**

    ii. Tailing dams **[ ]**

    iii. Others, specify…………………………………………………………………………………………………

22. How does mining companies manage their solid waste? ***(Please tick more than one if appropriate).***

    i. Recycling **[ ]**

    ii. Reuse **[ ]**

    iii. Landfill site **[ ]**

    iv. Open dump disposal **[ ]**

    v. Burning **[ ]**

    vi. Others, specify ……………………………………………………………………………………………………

23. What are the negatives effects associated with the following waste management practices by mining companies?

    i. Liquid waste management practices

    ………

    ………

    ………

    ii. Solid waste management practices

………

………

………

24. What is the level of noise produced by mining companies? Low **[ ],** Normal **[ ],** High **[ ]**
25. Does mining operations involve blasting? **Yes[ ]** **No[ ]**

    If yes, how **severe** is blasting noise and vibration? ***(Please tick one box for each).***

		**Low**	**Moderate**	**High**
I.	Blasting noise	[ ]	[ ]	[ ]
II.	Vibration	[ ]	[ ]	[ ]

26. Is there a noise pollution standard set for mining companies? **Yes [ ]** **No [ ]**

    If yes, does the emission of noise follow the set standard? ***(Please tick one).***

    I. Sometimes above standard **[ ]**
    II. Always within standard **[ ]**
    III. Always below standard **[ ]**

27. What is the quantities/amount **(decibels)** of noise emission by case company in the following years? 2015 **[ ],** 2016 **[ ],** 2017 **[ ],** 2018 **[ ],** 2019 **[ ]**
28. What are the operational activities that cause air pollution? ***(Please tick more than one if appropriate).***

    I. Fume/smoke from processing plants **[ ]**
    II. Smoke from vehicles and other sophisticated machines **[ ]**
    III. Dust from untarred roads use for transportation **[ ]**
    IV. Dust and chemicals released during blasting of extraction processes **[ ]**
    V. Excavation and Hauling of ore **[ ]**
    VI. HCN gas released from cyanide mixing **[ ]**
    VII. Others, specify…………………………………………………………………………………………………

29. How severe do these activities pollute the air? **(Please tick one box for each).**

**(Severity)**	**Low**	**Moderate**	**High**
Fume/smoke from processing plants	**[ ]**	**[ ]**	**[ ]**

Smoke from vehicles and other sophisticated machines [ ] [ ] [ ]

Dust from untarred roads for transportation [ ] [ ] [ ]

Dust and chemicals released during blasting or extraction processes [ ] [ ] [ ]

Others……

30. What are the remedial measures by mining companies to control the negative impacts their activities possess on the environment in the following areas ; Water, Air, Noise, Vegetation, Soil/Land?

Water pollution

………

………

………

………

………

………

………

Air pollution

………

………

………

………

………

………

………

………

Noise pollution

………

………

………

………

………

………

………

Soil/Land degradation

………

………

………

………

………

………

……..

31. What are some recommendations by the EPA/government to mining companies to help protect the environment and enhance environmental sustainability as mining operation unfold?

    ……

    ……

    ……

    ……

    ……..

    ……

    ……

    ……

    ……

    ……

Questionnaire for Environmental Officer of Case Company

TOPIC: Assessing the Environmental Impacts of Large-Scale Gold Mines, the Case of Nzema Gold Mines in the Ellembelle District of Ghana.

Note: ***This questionnaire is meant for academic and publications purposes with anonymity seriously observed.***

Questionnaire to help determine the Impacts of the activities of **LARGE SCALE** Gold mines on the environment of hosting communities (Nkroful, Anwia, T. Bokazo and Salman) and the District in general. The questionnaire is based on the following key elements of the environment: Water, Climate, Vegetation/Biodiversity, Air, Noise, and Soil/Land.

Kindly tick in the boxes and fills the spaces provided.

Age ………………   Gender ……………………..   Community ………………………….

Kindly tick in the boxes and fills the spaces provided.

32. How many large-scale mining companies operate the Nzema gold mines? …………….. and their name(s)………
33. What are the major water bodies (rivers and streams) around mining sites?

    ……………………………………………………………, ……………………………………………………………
    ……………………………………………………………, ……………………………………………………………
    ……………………………………………………………, ……………………………………………………………
    ……………………………………………………………, ……………………………………………………………
34. Which of these water bodies is/are affected by mining activities? ……
35. How are these water bodies affected by mining activities?

    ……
    ……

……

……

36. Does mining company(s) directly pump water from any of the available rivers for it operations? **Yes[ ]** **No[ ]**

    If yes, which river(s)……………………………………………………………………………………………………

37. Has there been any tailings dam (if any) leakage or seepage? **Yes[ ]** **No[ ]**
38. Has mining operations affected the climatic conditions of the District or host communities? **Yes[ ]** **No[ ]**
39. Does mining operations affect the rainfall pattern of the District? **Yes[ ]** **No[ ]**
40. If yes**,** how is the rainfall pattern affected? ***(Please tick more than one if appropriate).***

    iv) Increased in annual rainfall **[ ]**

    v) Decreased in annual rainfall **[ ]**

    vi) Constant changes in rainfall seasons **[ ]**

41. Does mining operations affect the temperature of the host communities? **Yes[ ]** **No[ ]**
42. If yes, how is temperature affected? ***(Please tick more than one if appropriate).***

    iv) Increasing/ high temperature over the year (annual) **[ ]**

    v) Decreasing/ low annual temperature **[ ]**

    vi) Constant variations in annual temperature **[ ]**

43. Has the mining activities or operations caused loss of vegetation cover? **Yes[ ]** **No[ ]**
44. What are the noticed negative effects of vegetation loss on lands and environment of host communities?

    ……

    ……

    ……

    ……

45. Are there abandoned mining sites in the district? **Yes[ ]** **No[ ]**
46. If yes, have there been afforestation or reclamation programs in abandoned mining sites? **Yes[ ]** **No[ ]**

47. Has the emergence of the mines caused changes in land-use patterns in hosting communities? **Yes[ ]** **No[ ]**
48. Does mining activities have effects on soil quality? **Yes[ ]** **No[ ]**

    If yes, how is the soil quality being affected by mining activities? ………
    ………
    ………
    ………
49. What is the PH level of soil in the hosting communities?

    Nkroful……………., Anwia……………., T.Bokazo…………………., Salman……………………
50. Has there changes in PH levels of soil as a result of the mining operations? **Yes[ ]** **No[ ]**
51. How does mining companies manage their liquid waste?

    iv. Discharge to water bodies **[ ]**

    v. Tailing dams **[ ]**

    vi. Others, specify……………………………………………………………………………………………
52. How does mining companies manage their solid waste? ***(Please tick more than one if appropriate).***

    vii. Recycling **[ ]**

    viii. Reuse **[ ]**

    ix. Landfill site **[ ]**

    x. Open dump disposal **[ ]**

    xi. Burning **[ ]**

    xii. Others, specify ……………………………………………………………………………………………………
53. What are the negatives effects associated with the following waste management practices by mining companies?

    iii. Liquid waste management practices

……

……

……

iv. Solid waste management practices

……

……

……

54. What is the level of noise produced by mining companies? Low **[ ],** Normal **[ ],** High **[ ]**

55. Does mining operations involve blasting? **Yes[ ]** **No[ ]**

If yes, how **severe** is blasting noise and vibration? ***(Please tick one box for each).***

		**Low**	**Moderate**	**High**
III.	Blasting noise	[ ]	[ ]	[ ]
IV.	Vibration	[ ]	[ ]	[ ]

56. Is there a noise pollution standard set for mining companies? **Yes [ ]** **No [ ]**

If yes, does the emission of noise follow the set standard? ***(Please tick one).***

IV. Sometimes above standard **[ ]**

V. Always within standard **[ ]**

VI. Always below standard **[ ]**

57. What is the quantities/amount **(decibels)** of noise emission by case company in the following years? 2015 **[ ],** 2016 **[ ],** 2017 **[ ],** 2018 **[ ],** 2019 **[ ]**

58. What are the operational activities that cause air pollution? ***(Please tick more than one if appropriate).***

VIII. Fume/smoke from processing plants **[ ]**

IX. Smoke from vehicles and other sophisticated machines **[ ]**

X. Dust from untarred roads use for transportation **[ ]**

XI. Dust and chemicals released during blasting of extraction processes **[ ]**

XII. Excavation and Hauling of ore **[ ]**

XIII. HCN gas released from cyanide mixing **[ ]**

XIV. Others, specify………………………………………………………………………………………

59. How severe do these activities pollute the air? **(Please tick one box for each).**

(Severity)	Low	Moderate	High
Fume/smoke from processing plants	[ ]	[ ]	[ ]
Smoke from vehicles and other sophisticated machines	[ ]	[ ]	[ ]
Dust from untarred roads for transportation	[ ]	[ ]	[ ]
Dust and chemicals released during blasting or extraction processes	[ ]	[ ]	[ ]

Others……

60. What are the remedial measures by mining companies to control the negative impacts their activities possess on the environment in the following areas ; Water, Air, Noise, Vegetation, Soil/Land?

Water pollution

……

……

……

……

……

……

……

Air pollution

……

……

……

……

……

………

………

……….

Noise pollution

………

………

………

………

………

………

………

Soil/Land degradation

………

………

………

………

………

………

…….

__Questionnaire for Farmers__

TOPIC: Assessing the Environmental Impacts of Large-Scale Gold Mines, the Case of Nzema Gold Mines in the Ellembelle District of Ghana.

Note: ***This questionnaire is meant for academic and publications purposes with anonymity seriously observed.***

Questionnaire to help determine the Impacts of the activities of **LARGE SCALE** Gold mines on the environment of hosting communities (Nkroful, Anwia, T. Bokazo and Salman) and the District in general. The questionnaire is based on the following key elements of the environment: Water, Climate, Vegetation/Biodiversity, Air, Noise, and Soil/Land.

Kindly tick in the boxes and fills the spaces provided.

Age ……………… Gender …………………….. Community ………………………….

1. What type of farming system do you practice? **Commercial [ ] Subsistence [ ]**
2. What type of crop do you cultivate? **Cash crop [ ] Food crop [ ] Both [ ]**
3. What is the estimated acre of your farm land? **1-5 [ ] 6-10 [ ] 10-15 [ ] 15+[ ]**
4. How close is your farm to mining sites? **<1km [ ] 1-2km [ ] 2-4km [ ] 5-7km[ ] 8+km [ ]**
5. Has the mining company claimed/taken part of your farmland? **Yes [ ] No [ ]**
6. If yes, how many acres were taken? **<half of total land [ ] ≥half of total land [ ] All [ ]**
7. Has mining activities shortened agriculture land use fallows? Yes [ ] No [ ]
8. Does mining activities affect your faming activities or output? **Yes [ ] No [ ]**
9. If yes, how does it affect your farming?

   ………

   ………

   ………
10. Have you reported these impacts to any authorities? **Yes [ ] No [ ]**
11. If yes, who did you reported to? **Mine social responsibility office(r) [ ] Community elders [ ] District assembly [ ]**
12. How was the response after your report?

    ………

    ………

13. Is there any water body (rivers and streams) closer to your farm for agricultural purposes?

    Yes [ ] **No [ ]**

14. If yes, what is the name of the river(s) or streams?

    ………

15. Is this river or stream polluted or affected by mining activities? **Yes [ ]** **No [ ]**

16. If yes, how is it polluted?

    Direct discharge of mining waste **[ ]**

    Tailings dam spillage **[ ]**

    Effluent from mine sites **[ ]**

    Continues pumping of water from the river **[ ]**

    Redirecting/ diverting the direction of flow of river or stream **[ ]**

    Filling streams with huge rocks causing it shrinkage [**]**

    Others, specify……

17. Has operations caused loss of vegetation cover around your community? **Yes [ ]** **No[ ]**

18. What are the noticed negative effects of vegetation loss on lands and ecosystem?

    ………

    ………

    ………

    ………

19. Is your occupation group (Farmers) considered during environmental and mining related decision making processes? **Yes [ ]** **No [ ]**

20. Does your community receive information education from stakeholder institutions (EPA, District Assembly, Forestry, and Case Company) concerning the environment and other mining related issues? **Yes [ ]** **No [ ]**

21. If yes, how often are these educations given in the community?

    Very seldom **Seldom** **Often** **Very often** **(***please select 1)*

    [ ] **[ ]** **[ ]** **[ ]**

22. How do you see the corporations amongst stakeholder institutions in discharging their duties?

    Very weak **Weak** **Strong** **Very strong** ***(please select 1)***

    [ ] **[ ]** **[ ]** **[ ]**
